# Asymptomatic detection of SARS‐CoV‐2 among cancer patients receiving infusional anti‐cancer therapy

**DOI:** 10.1002/cam4.4373

**Published:** 2021-10-28

**Authors:** Justin Shaya, Angelo Cabal, Taylor Nonato, Francesca Torriani, Joseph Califano, Scott Lippman, Assuntina Sacco, Rana R. McKay

**Affiliations:** ^1^ University of California San Diego Moores Cancer Center La Jolla California USA; ^2^ Division of Infectious Disease University of California San Diego La Jolla California USA

**Keywords:** asymptomatic, cancer, Covid‐19, PCR testing, screening

## Abstract

**Background:**

Little is known regarding the rate and clinical outcomes of asymptomatic carriers of SARS‐CoV‐2 among patients with cancer. Detection of asymptomatic carriers is important in this population given the use of myelosuppressive and immunomodulating therapies. Understanding the asymptomatic carrier rate will help to develop mitigation strategies in this high‐risk cohort.

**Methods:**

Retrospective cohort analysis of an asymptomatic screening protocol which required patients receiving infusional anti‐cancer therapy to undergo a symptom/exposure screen and SARS‐CoV‐2 PCR testing 24–96 h prior to their infusion. The primary outcome of this analysis was the rate of asymptomatic SARS‐CoV‐2 infection. Secondary outcomes included the rate of COVID‐19‐related hospitalization and mortality and delays in oncologic therapy.

**Results:**

Among a cohort of 2691 cancer patients who underwent asymptomatic screening, 1.6% (*N* = 43/2691) of patients were found to be SARS‐CoV‐2 positive on asymptomatic screening. 11.6% (*N* = 5/43) of the cohort ultimately developed COVID‐19‐related symptoms. Four patients required hospitalization for complications of COVID‐19 infection. No patient died from COVID‐related complications. 97.7% (*N* = 42/43) had their anti‐cancer therapy delayed or deferred with a median delay of 21 days (range: 7–77 days).

**Conclusions:**

Overall, among a cohort of active cancer patients receiving anti‐cancer therapy, an asymptomatic SARS‐CoV2 PCR‐based screening protocol detected a small cohort of asymptomatic carriers. The majority of these patients remained asymptomatic on long‐term follow‐up and outcomes were much more favorable compared to previously described outcomes of cancer patients with symptomatic COVID‐19 infection.

## Graphical abstract

Little is known about the rate of asymptomatic SARS‐CoV‐2 carriers among cancer patients. Here, we examined a cohort of active cancer patients who underwent asymptomatic SARS‐CoV‐2 PCR screening prior to their infusion therapy. We found a low rate of asymptomatic carriers (1.6%) among this cohort and this asymptomatic cohort had favorable longterm infectious outcomes.

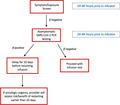

## 1 INTRODUCTION

The SARS‐CoV‐2 pandemic has significantly impacted patients with cancer and delivery of cancer care. Large cohort studies have shown that patients with cancer have significant rates of mortality with COVID‐19 infection.^1^, ^2^, ^3^, ^4^ Among this heterogenous cohort, patients with active cancer have mortality estimates as high as 30%.^3^, ^4^, ^5^ This has required optimization of oncologic care delivery to mitigate COVID‐19 risk.

Patients receiving active anti‐cancer therapy represent a high‐risk group. Given this, an important aspect of cancer care delivery has been the development of screening strategies for patients prior to initiation of systemic therapy. Detection of SARS‐CoV‐19 prior to receipt of potentially immunosuppressive and/or immunomodulatory therapies may help mitigate morbidity associated with COVID‐19 infection. Additionally, infusion centers are often shared spaces with multiple patients receiving treatment at a given time. Detection and isolation of SARS‐CoV‐19 infected patients may aid in reducing spread to other high‐risk patients.^6^ Multiple strategies have been utilized including masking and physical distancing, symptom and exposure screening, visitor restriction policies, and systematic PCR screening of high‐risk individuals.^6^, ^7^, ^8^ The ability to perform PCR testing on asymptomatic individuals has been limited by testing availability.

Little is known about the rates of asymptomatic carriers among patients with cancer. Several retrospective studies among patients receiving anti‐cancer therapies have noted low rates of asymptomatic carriers ranging from 0.57%–0.74%.^7^, ^9^ At UC San Diego Health, given the rapid expansion of SARS‐CoV‐2 PCR testing, an asymptomatic screening protocol for cancer patients receiving infusional anti‐cancer therapies was implemented. With this screening protocol, patients were required to undergo a COVID‐19 screening questionnaire and if asymptomatic, SARS‐CoV‐2 PCR testing 24–96 h prior to infusion. Understanding the prevalence of asymptomatic SARS‐CoV‐2 infection and outcomes among this population is critical to developing optimal screening strategies for cancer patients during the pandemic.

In this analysis, we report on the results of this asymptomatic screening protocol for cancer patients receiving infusional anti‐cancer therapies and outcomes of SARS‐CoV‐2 carriers with cancer. The primary objective of this study is to assess the rate of asymptomatic SARS‐CoV‐2 infection among an active cancer cohort.

## 2 METHODS

**FIGURE 1:**
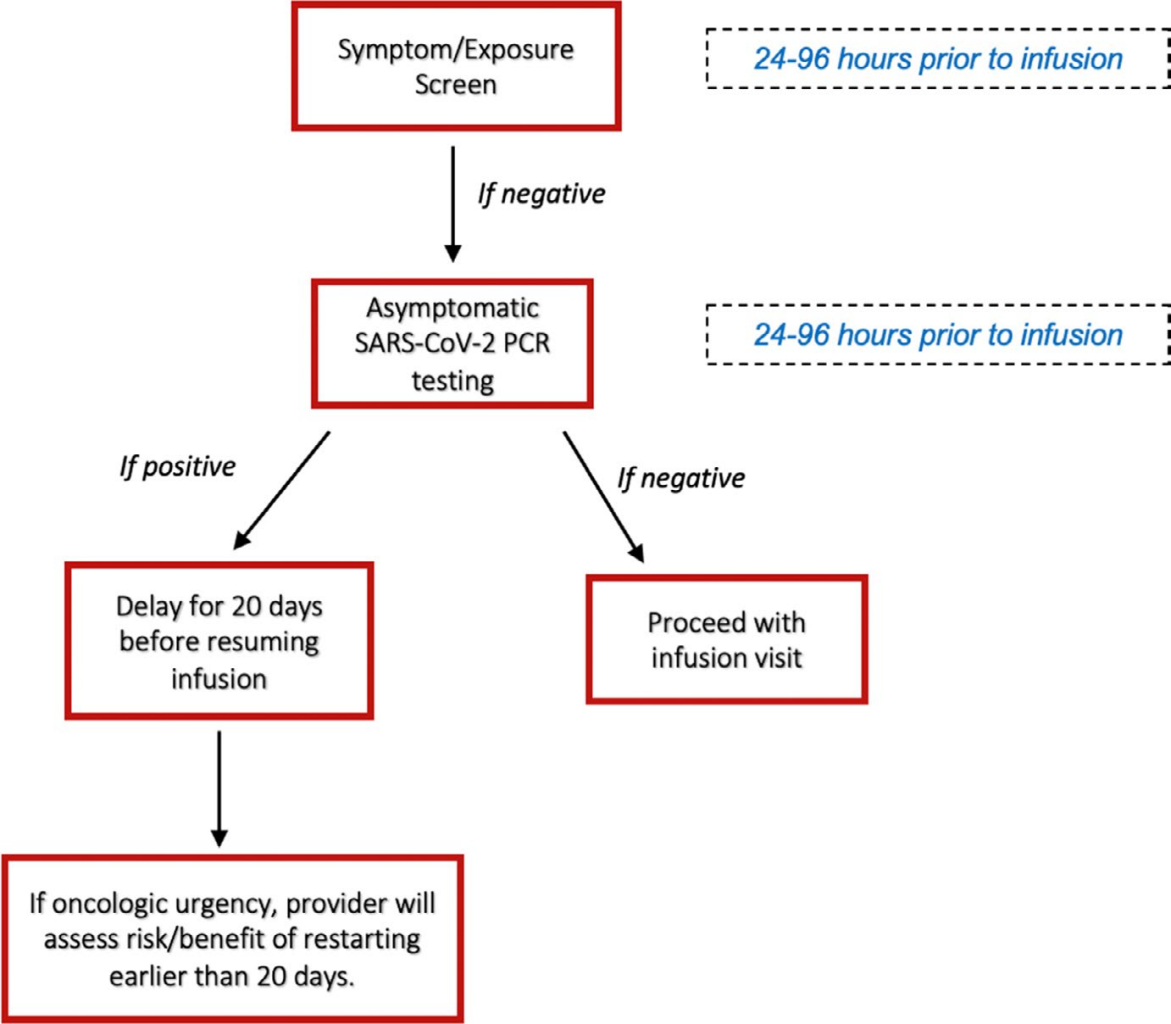
UC San Diego health asymptomatic testing strategy for cancer patients

This was a single‐center analysis of an asymptomatic COVID‐19 screening protocol of patients with active cancer receiving infusional anti‐cancer therapy between June 1, 2020 and February 15, 2021 at UC San Diego Health System (Figure 1). All cancer patients treated with an infusional anti‐cancer therapy were required to undergo both a COVID‐19 symptom/exposure questionnaire, and if negative, an asymptomatic SARS‐CoV‐2 PCR test 24–96 h prior to infusion. Patients undergoing recurrent infusions were tested every 3–6 weeks prior to each infusion. Patients were excluded from this analysis if they had symptoms related to suspected or proven COVID‐19 disease. Patients were consecutively identified using the electronic medical record.

If an asymptomatic patient tested positive for SARS‐CoV‐2, the protocol recommended that the infusion be delayed for 20 days. If the patient remained asymptomatic, therapy could be resumed after day 20. Delays in therapy were at the discretion of the treating physician. Therapy could resume earlier than 20 days, if indicated per the treating physician, with isolation procedures in the infusion center. Repeat PCR testing was initially included as an option to discontinue isolation precautions, but this was eliminated in July 2020 in favor of a symptom‐based approach.

The primary endpoint of the study was the rate of SARS‐CoV‐2 infection among asymptomatic patients. Secondary endpoints included COVID‐19‐related outcomes including the proportion of asymptomatic patients requiring hospitalization and COVID‐19‐related mortality. Patterns of oncologic management for asymptomatic SARS‐CoV‐2 positive patients were described including duration of delays in therapy and indications to resume therapy. Baseline characteristics were quantitatively described and for each patient, the Charlson Comorbidity Index (CCI) was calculated at the time of positive SARS‐CoV‐2 test.^13^ This study was Institutional Review Board approved.

## 3 RESULTS

A cohort of 2691 cancer patients was identified that received infusional anti‐cancer therapy and underwent at least 1 asymptomatic SARS‐CoV‐2 PCR test during the study period. Among this cohort, 1.6% (*N* = 43/2691) (95% CI: 1.2%–2.2%) of patients were found to be SARS‐CoV‐2 positive on asymptomatic screening (Table 1). Of the 43 positive patients, 18.6% (*N* = 8/43) had hematologic malignancies and 81.4% (*N* = 35/43) had solid tumors. At the time of positive testing, 65.1% (*N* = 28) were receiving cytotoxic chemotherapy, 16.2% (*N* = 7) immunotherapy, 11.6% (*N* = 5) targeted therapy, and 7.0% (*N* = 3) were on a clinical trial. The majority of patients were diagnosed between December 1, 2020 and February 15, 2021. During the study period, an additional 89 patients (3.5%) of the 2691 patients were diagnosed with symptomatic COVID‐19 infection outside of the screening protocol. The median CCI score of the cohort was 6.

**TABLE 1:** Baseline characteristic of asymptomatic COVID‐19 positive cohort

Characteristic	*N* (*N*%); *N* = 43 total
Age, median (range)	55 (24–81)
Female sex	28 (65.1%)
Type of malignancy	
Solid tumor	35 (81.4%)
Localized	8 (18.6%)
Metastatic	11 (25.6%)
Hematologic malignancy	8 (18.6%)
Type of therapy received	
Cytotoxic chemotherapy	28 (65.1%)
Immunotherapy	7 (16.2%)
Targeted therapy	5 (11.6%)
Clinical trial	3 (7.0%)
ECOG, median (range)	1 (0–2)
History of allogenic or autologous stem cell transplant	0 (0%)
Chronic use of immunosuppressive medications	1 (2.3%)
Charlson Comorbidity Index score at time of SAR‐CoV−2 diagnosis, median (range)	6 (2–12)
Comorbidities of cohort	
Cardiovascular disease	19 (44.2%)
Chronic respiratory disease	2 (4.7%)
Liver disease	1 (2.3%)
Metabolic disease	9 (20.9%)
Renal disease	1 (2.3%)
Date of positive SARS‐CoV−2 PCR test	
June 1, 2020 to August 31, 2020	13 (30.2%)
September 1, 2020 to November 30, 2020	8 (18.6%)
December 1, 2020 to February 15, 2021	22 (51.2%)

With a median follow‐up of 89 days from SARS‐CoV‐2 positive testing (range: 10–274 days), 11.6% (*N* = 5/43) of patients ultimately developed COVID‐19‐related symptoms. Median time from testing to symptoms was 7.5 days (range: 4–10). Among the five symptomatic patients, four required hospitalization for complications of COVID‐19 infection. Median time to hospitalization from initial SARS‐CoV‐2 test was 8 days (range: 4–10 days). Among the four hospitalized patients, three required supplemental oxygen and no patient required intubation. No patient died from COVID‐related complications.

**FIGURE 2:**
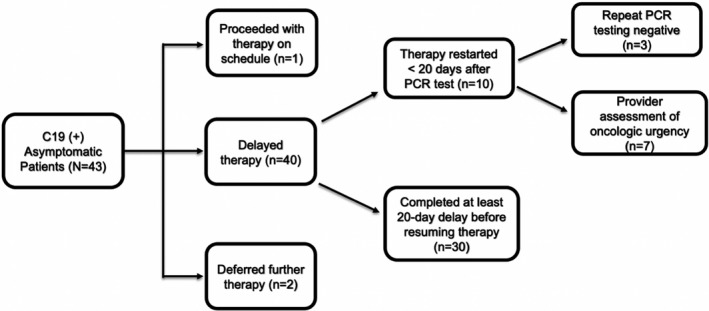
Oncologic management of asymptomatic cohort

With regards to oncologic outcomes, given that testing was done prior to infusion, 97.7% (*N* = 42/43) had their anti‐cancer therapy delayed or deferred with a median delay of 21 days (range: 7–77 days) (Figure 2). Among the cohort, only 1 patient proceeded with cytotoxic chemotherapy on schedule in the setting of adjuvant chemoradiation for squamous cell carcinoma of the oropharynx.

## 4 DISCUSSION

The COVID‐19 pandemic has presented unique obstacles in cancer care and the need to develop novel strategies to mitigate risks among high‐risk cancer patients. An improved understanding of the rates of asymptomatic SARS‐CoV‐2 carriers helps to inform the ideal screening strategy. The asymptomatic screening protocol described provides a unique platform to assess the rate of asymptomatic COVID‐19 carriers with a defined denominator of patients given the institutional requirement that all patients undergo this screening prior to infusion.

In this study, we demonstrate a low rate of asymptomatic carriers of SARS‐CoV‐2 infection with a rate of 1.6%. This rate is somewhat higher than previously described smaller cohort analyses.^7^, ^9^ While the majority of patients remained asymptomatic with sufficient follow‐up time, 11.6% (*N* = 5) of patients ultimately developed symptoms and COVID‐19 testing in these cases likely detected pre‐symptomatic cases. These findings support that a symptom‐exposure screen may be insufficient to detect all COVID‐19 cases, and that if available, the addition of routine PCR screening provides more comprehensive detection.

While the addition of PCR‐based screening provides robust assessment of asymptomatic carriers, there are limitations. Screening all patients with PCR testing is associated with significant cost and the overall cost‐effectiveness of this strategy remains unclear, particularly given the low numbers of positive cases. Moreover, this protocol was made possible by sufficient access to PCR testing, and given issues with access to testing throughout the pandemic, this type of protocol may not be feasible at other centers. As the pandemic continues, the optimal screening strategy for cancer patients continues to be refined and will depend on the capabilities of each individual region and practice.

It is currently unknown what factors are associated with asymptomatic SARS‐CoV2 infection. In assessing for established risk factors for COVID‐19 infection,^1^ it was notable that among the asymptomatic cohort, patients were younger, the majority were female with solid tumors, and the median ECOG was 1. All of these characteristics have been associated with improved outcomes in cancer patients infected with SARS‐CoV2.^1^, ^2^, ^3^, ^4^ With regards to comorbidities, the majority of the cohort had few comorbidities and the median CCI was 6, with this score being driven largely by the fact that all patients in this cohort had a cancer diagnosis. Given the small size of the cohort, further stratification of outcomes by clinical characteristics was not possible. Future comparative analysis between asymptomatic and symptomatic cohorts of cancer patients with COVID‐19 infection will be important in identifying risk factors for symptomatic COVID‐19 infection.

Lastly, asymptomatic carriers in this cohort appeared to have favorable long‐term infectious outcomes with few developing symptoms and even fewer requiring hospitalization. The effect of anti‐cancer therapies on COVID‐19 severity and mortality remains unclear with mixed data on the impact of anti‐cancer therapies on COVID‐19 severity and mortality.^2^, ^3^, ^12^ Here, the majority of patients underwent the recommended delay in therapy and only one patient proceeded with therapy without delay. Further work is ultimately needed to understand the underlying biology of asymptomatic SARS‐CoV2 carriers to allow for better prediction and risk stratification of these cohorts.

## 5 CONCLUSION

In conclusion, the rate of asymptomatic SARS‐CoV‐2 cases among cancer patients receiving active therapy was found to be 1.6%, with the majority of patients remaining asymptomatic on long‐term follow‐up. Further work is ultimately needed to understand the underlying biology of asymptomatic SARS‐CoV2 carriers to allow for better prediction and risk stratification of these cohorts.

## CONFLICT OF INTEREST

RRM received research funding from Bayer, Pfizer, Tempus; serves on Advisory Board for AstraZeneca, Bayer, Bristol Myers Squib, Calithera, Exelixis, Janssen, Merck, Novartis, Pfizer, Sanofi, Tempus; is a consultant for Dendreon, Myovant, Vividion. All unrelated to the present work.

## ETHICS STATEMENT

This work was approved by the Institutional Review Board at the University of California San Diego.

## Data Availability

Deidentified analytical data will be made available upon request to the corresponding author.
